# Antimicrobial Activity of UV-Activated and Cysteamine-Grafted Polymer Foils Against Bacteria and Algae

**DOI:** 10.3390/polym17020251

**Published:** 2025-01-20

**Authors:** Viktorie Neubertová, Tereza Silovská, Václav Švorčík, Zdeňka Kolská

**Affiliations:** 1 Centre for Nanomaterials and Biotechnology, Faculty of Science, University of Jan Evangelista Purkyně, Pasteurova 15, 400 96 Ústí nad Labem, Czech Republic; tknapova@seznam.cz (T.S.); zdenka.kolska@ujep.cz (Z.K.); 2 Department of Solid State Engineering, Faculty of Chemical Technology, University of Chemistry and Technology, Technická 5, 166 28 Prague, Czech Republic; vaclav.svorcik@vscht.cz

**Keywords:** antimicrobial activity, chemical grafting, polymer foils, UV radiation, zeta potential

## Abstract

Surface modification of various polymer foils was achieved by UV activation and chemical grafting with cysteamine to improve surface properties and antimicrobial efficacy. UVC activation at 254 nm led to changes in surface wettability and charge density, which allowed the introduction of amino and thiol functional groups by cysteamine grafting. X-ray photoelectron spectroscopy (XPS) confirmed increased nitrogen and sulfur content on the modified surfaces. SEM analysis revealed that UV activation and cysteamine grafting resulted in distinct surface roughness and texturing, which are expected to enhance microbial interactions. Antimicrobial tests showed increased resistance to algal growth (inhibition test) and bacterial colonization (drop plate method), with significant improvement observed for polyethylene terephthalate (PET) and polyetheretherketone (PEEK) foils. The important factors influencing the efficacy included UV exposure time and cysteamine concentration, with longer exposure and higher concentrations leading to bacterial reduction of up to 45.7% for *Escherichia coli* and 55.6% for *Staphylococcus epidermidis*. These findings highlight the potential of combining UV activation and cysteamine grafting as an effective method for developing polymeric materials with enhanced antimicrobial function, offering applications in industries such as healthcare and packaging.

## 1. Introduction

Polymers are widely used in industries ranging from healthcare to packaging due to their durability, light weight, and chemical resistance [1,2,3,4]. However, their limited surface wettability and lack of inherent antimicrobial properties restrict their use in applications where surface interactions are important [1,5]. Recent advances in polymer surface modification aim to overcome these limitations and make polymers more adaptable for antimicrobial use and biocompatibility without changing their bulk properties. Techniques such as plasma treatment and laser modification [1,2,3,4,5,6,7,8,9,10,11], chemical modifications [12,13,14], and UV irradiation [15,16,17,18,19,20,21,22,23] have attracted attention due to their effectiveness in creating reactive sites on polymer surfaces for further chemical modification. These methods can be applied either individually or in combination [5,10,11,17,24]. Among these techniques, UV radiation represents an affordable and efficient approach that creates reactive sites without the need for complex conditions or high-energy processes as in plasma activation [11,16]. UV light initiates bond cleavage on polymer surfaces, creating reactive sites for chemical grafting [15,22]. Adjusting UV wavelength, exposure time, and intensity can influence the chemical changes induced on the polymer surface [25,26]. Notably, UV radiation is often used to modify synthetic polymers for biomedical applications [27,28,29].

One promising advancement involves the grafting of pre-activated polymer surfaces with chemicals containing various functional groups [30]. Cysteamine, a small organic molecule containing amino and thiol functional groups, is highly reactive [31]. Due to its antibacterial properties, cysteamine is advantageous for chemical grafting in antimicrobial applications [32,33]. The thiol groups in cysteamine disrupt bacterial cell membranes and interfere with microbial metabolic processes, enabling surfaces to actively inhibit bacterial growth and adhesion [34,35]. This antibacterial activity makes cysteamine valuable for creating surfaces that prevent bacterial growth and adhesion. Antimicrobial surfaces are increasingly important as bacteria become more resistant to common antibacterial agents [36,37]. An ideal antibacterial system would efficiently combat a broad spectrum of bacteria at the lowest effective concentration.

Chemical grafting of polymeric materials with cysteamine shows great potential in the development of antibacterial surfaces [38,39,40,41]. Recent studies have focused on the modification of various polymeric materials by chemically grafting cysteamine onto them. For example, amine-functionalized polyester dendrimers have been synthesized to overcome the problems associated with rapid depolymerization under physiological conditions and to achieve enhanced stability and antibacterial activity [42,43]. In particular, the second-generation dendrimers functionalized with cysteamine showed strong antibacterial efficacy and minimal toxicity, making them promising for biomedical applications. Similarly, self-immolative poly(benzyl ether)s (PBEs) were developed with pendant cationic ammonium groups and grafted PEG side chains using post-polymerization thiol–ene chemistry [44]. PBEs containing 50% cysteamine and 50% PEG-800 side chains displayed remarkable cell selectivity (HC50/MBC ~28) and superior antibacterial performance compared to conventional homopolymers. Additionally, amphiphilic cationic polysiloxanes (PDMS-g-AH) were prepared via thiol–ene “click” chemistry and modified with cysteamine, achieving improved stability, tunable molecular weights, and low biotoxicity [35]. These materials demonstrated versatile antimicrobial properties against bacteria and fungi, showcasing the influence of grafting ratios and molecular design on their performance. Previous studies have shown that grafting cysteamine onto polymer surfaces can enhance antibacterial properties, yet there is limited understanding of how different polymer structures affect grafting efficiency and antimicrobial outcomes.

This study investigates a range of polymers, including polyethylene terephthalate (PET), polytetrafluoroethylene (PTFE), ultra-high-molecular-weight polyethylene (UHMWPE), polyvinylidene chloride (PVDC), polypropylene (PP), polystyrene (PS), unplasticized polyvinyl chloride (UPVC), polyvinylidene fluoride (PVDF), polyoxymethylene (POM), and polyetheretherketone (PEEK), to better understand how material composition influences the effects of UV-activated cysteamine grafting. Building on our previous work [22], which focused only on PET and PEEK modified with amino acids and curcumin, this study broadens the investigation by systematically testing cysteamine at varying concentrations (10 wt. %, 20 wt. %, and 30 wt. %) across a wider range of materials. By testing various UV activation parameters and cysteamine concentrations, this study aims to provide comparative insights that may assist in selecting materials and adapting surface modifications for different applications. To test the broad-spectrum antimicrobial properties of the modified polymers, we selected three representative microbes. *Escherichia coli* (*E. coli*) was chosen to represent Gram-negative bacteria, which often show higher resistance to antimicrobials. *Staphylococcus epidermidis* (*S. epidermidis*) was selected as a Gram-positive bacterium commonly involved in surface biofilm formation. In addition, this study introduces *Desmodesmus quadricauda* (*D. quadricauda*), a freshwater algal species, to assess antifouling properties, expanding the range of applications explored beyond those considered in previous work. Advanced characterization techniques, such as scanning electron microscopy (SEM) and X-ray photoelectron spectroscopy (XPS), were used in this study. These methods complement goniometry and electrokinetic analysis by providing direct evidence of surface changes previously inferred from contact angle and zeta potential measurements. These findings aim to support future research and development of antimicrobial and antifouling materials for applications in healthcare, packaging, and environmental protection.

## 2. Materials and Methods

### 2.1. Materials

The substrates under study included a range of polymeric foils of varying polarity, sourced from GoodFellow, Huntingdon, UK. The following materials were examined: PET (50 μm), PTFE (50 μm), UHMWPE (75 μm), PVDC (43 μm), PP (50 μm), PS (50 μm), UPVC (50 μm), PVDF (50 μm), POM (500 μm), and PEEK (50 μm). For the activation of the polymer foil surfaces, a UV lamp with a power rating of 40 W emitting at a wavelength of 254 nm was used. Subsequently, for the chemical grafting step, cysteamine (CYS) (HS(CH_2_)_2_NH_2_, 98%, Sigma Aldrich, St. Louis, MI, USA) was used as the reagent.

### 2.2. Surface Activation and Chemical Modification of the Polymer Foils

Polymer foils underwent surface activation at room temperature (RT) using a UV lamp with a power rating of 40 W emitting at wavelength of 254 nm (UVC). Subsequently, chemical grafting was performed with cysteamine (CYS) water solutions at different concentrations (10, 20 or 30 wt. %) through three different procedures: (i) CYS solutions of varying concentrations were directly grafted onto polymer surfaces without prior UV radiation activation. This chemical grafting was realized under UV radiation for 10, 30, and 60 min. (ii) Samples were first pre-activated under the UV lamp for 10, 30, or 60 min. Following pre-activation, the samples were immersed in CYS solutions with different concentrations for 60 min, but outside the influence of UV radiation. (iii) Polymer foils were activated by UV radiation for 10, 30, or 60 min using a UV lamp. Subsequently, the activated samples were immersed in cysteamine solutions of varying concentrations for 60 min under the same UV lamp. Importantly, it was observed that all three procedures yielded similar results in terms of changes in surface properties. Thus, in order to be concise and relevant, the study focuses mainly on the outcomes gained from the process (ii): the pre-activation of polymer foils under UV radiation, followed by grafting with cysteamine at different concentrations. A summary of sample markings is presented in Table 1.

### 2.3. Characterization Methods

Surface wettability was determined by measuring the static contact angle of distilled water at RT using the DSA30 instrument (Krüss, Hamburg, Germany) and the sessile drop method. A 2.0 ± 0.1 µL volume of deionized water was deposited by a microneedle, and each sample was measured five times at different positions. The Advance program was utilized to evaluate the static contact angle with a 5% margin of error.

Electrokinetic analysis, including zeta potential determination, was conducted using the SurPASS electrokinetic analyzer (Anton Paar, Graz, Austria). Two samples of the same surface, each sized at 2 × 1 cm^2^, were fixed on sample holders. Measurements were performed in a cell with an adjustable gap of approximately 100 µm at RT, atmospheric pressure, and a constant pH of 6.7, with an experimental error of 5%. Zeta potential was determined using the streaming current method, and the Helmholtz–Smoluchowski equation was applied to calculate zeta potential.

Surface elemental analysis was carried out using X-ray photoelectron spectroscopy (Omicron Nanotechnology ESCAProbe spectrometer, Omicron Nanotechnology GmbH, Taunusstein, Germany). The detection angles of secondary photoelectrons were set at 90° from the surface normal. The X-ray source was monochromated at 1486.7 eV. The exposed and examined area had dimensions of 2 × 3 mm^2^. Survey spectra were recorded with a pass energy of 40 eV and a step size of 0.5 eV. The regions of detected elements were further analyzed with a pass energy of 10 eV, repeated ten times with a step size of 0.1 eV. Spectra were referenced to the C1s peak of aliphatic C–C bonds at 285 eV. Data from 10 eV scans were used for quantification, and data processing was performed using CasaXPS software (version 2.3.16).

Scanning electron microscopy (SEM) was performed using a Vega II LSH microscope (Tescan, Brno, Czech Republic) equipped with a secondary electron (SE) detector. The foil samples were fixed onto sample holders using double-sided carbon tape and sputter-coated with a thin (10 nm) gold layer to improve conductivity. The micrographs were processed and analyzed using VegaTC software (version 2.1).

The antimicrobial behavior of the prepared samples was examined through two different tests: (i) a test of grown inhibition in *D. quadricauda* algae and (ii) antibacterial tests using the drop plate method with *E. coli* (Gram-negative bacteria, DBM 3138) and *S. epidermidis* (Gram-positive bacteria, DBM 2124). Selected strains were obtained from the collection of microorganisms at the Department of Biochemistry and Microbiology of the University of Chemistry and Technology (Prague, Czech Republic).

Polymer foils were immersed in physiological saline solution. A single colony-forming unit (CFU) of each bacterial strain was inoculated into Luria–Bertani (LB) medium and cultivated on an orbital shaker at 37 °C for 16 h to prepare the bacterial suspensions. The cultures were then serially diluted in sterile phosphate-buffered saline (PBS, pH 7.4) to achieve a final concentration of 1.0 × 10^4^ CFU mL^−1^ for *E. coli* and 4.0 × 10^4^ CFU mL^−1^ for S. epidermidis. The polymer samples were incubated in 2 mL of the bacterial suspension at laboratory temperature under static conditions for 2 h. Bacterial suspensions incubated only in pure physiological saline solutions served as controls. Aliquots of 25 μL from all samples were placed on LB agar plates, and the growth of colonies was evaluated after 20 h at 37 °C for *S. epidermidis* and at RT for *E. coli*. Each sample was prepared separately in triplicate under sterile conditions.

The algal growth inhibition test was carried out based on an adopted procedure following [45] with *D. quadricauda* under continuous illumination for all samples under study. An algal suspension (2.5 mL) was dropped onto the surface of polymer foil (1 × 3 cm^2^) in Petri dishes. The concentration of algae was determined by direct counting under a microscope in a counting chamber (Cyrrus II). The first sample was withdrawn (~0.1 mL) one hour after suspension addition, followed by measurements after one day and one week. All samples were evaluated ten times. The algal growth inhibition test results were evaluated based on the mean values calculated from ten independent measurements for each sample.

## 3. Results and Discussion

### 3.1. Surface Wettability Measurements

**Figure 1:**
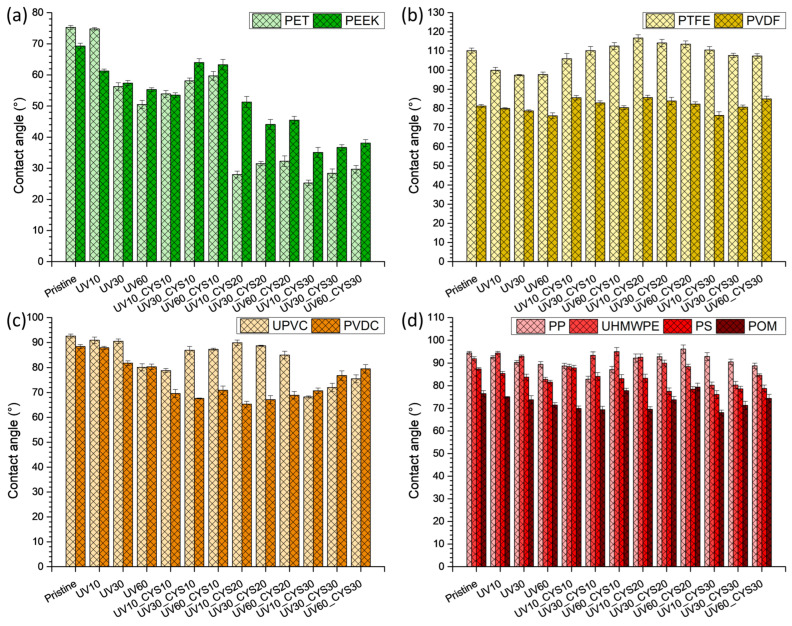
Comparison of contact angle values of all pristine and functionalized polymers under study: (**a**) PET and PEEK; (**b**) PTFE and PVDF; (**c**) UPVC and PVDC; (**d**) PP, UHMWPE, PS, and POM.

Surface wettability is an important factor in determining how materials interact with their environment, particularly in applications where increased hydrophilicity can improve performance [46,47,48]. Contact angle measurements presented in Figure 1 were used to evaluate the effects of UV activation and cysteamine grafting on polymer surfaces.

The analysis of contact angle measurements for PET and PEEK samples (Figure 1a) revealed significant changes in surface wettability following UV activation and subsequent cysteamine grafting. The pristine samples of both PET and PEEK had initial contact angles indicating moderate hydrophilicity. For PET, the pristine sample showed a contact angle of 75.3° ± 0.6°, while PEEK exhibited an angle of 69.3° ± 0.9°, which aligns with their inherent surface properties. Upon UV treatment (samples UV10, UV30, and UV60), a clear trend of decreasing contact angles was observed. PET samples treated with UV for varying durations showed reductions in contact angles from 74.8° ± 0.4° to 50.5° ± 1.3°, suggesting an increase in surface hydrophilicity due to the formation of polar functional groups such as hydroxyl and carbonyl groups. Similarly, PEEK samples displayed a reduction from 69.3° ± 0.9° to 55.3° ± 0.6°, highlighting that UV exposure effectively activates these surfaces by introducing reactive sites. The impact of cysteamine grafting was even more pronounced, especially at higher concentrations. For PET, UV10_CYS20 showed a substantial decrease in contact angle to 28° ± 1.1°, indicating significant surface modification and improved hydrophilicity. This effect can be attributed to the grafting of cysteamine, which introduces amino and thiol groups that enhance surface polarity. PEEK samples also demonstrated notable reductions in contact angle with cysteamine treatment, reaching a value of 51.3° ± 1.8° for UV10_CYS20 and an even lower value of 35.1° ± 1.6° for UV10_CYS30, where the cysteamine concentration was maximized. These preliminary results illustrate the response of PET and PEEK to UV activation and cysteamine grafting for a broader analysis of how different polymer types respond to similar treatments [49].

Moving on to Figure 1b, the contact angle variations for PTFE and PVDF are presented. Following UV irradiation, a subtle decrease in contact angle values can be observed compared to pristine samples. Subsequent cysteamine grafting induces a slight increase in contact angle compared to irradiated samples, signifying a modification of surface properties. Notably, unlike PET and PEEK, these fluorine-containing polymers, when treated with cysteamine, exhibit increased hydrophobicity. This distinction underscores the different responses of PTFE and PVDF to UV light and cysteamine grafting, emphasizing the influence of their fluorine-rich composition on wettability.

Examining UPVC and PVDC in Figure 1c reveals contact angle changes similar to PTFE and PVDF after UV treatment, with both polymers showing a slight decrease. However, after grafting with a 10% or 20% cysteamine concentration, UPVC surfaces become more hydrophobic than after UV treatment, contrasting with PVDC, where hydrophobic changes are less evident. We attribute the differences to the chlorine content in their structures. The higher chlorine content of PVDC contributes to a more stable and less reactive surface, potentially affecting the extent of hydrophobic modifications. Conversely, the lower chlorine content of UPVC offers more reactive sites for cysteamine grafting, resulting in more pronounced changes in hydrophobicity.

Finally, Figure 1d illustrates contact angle results for other polymers, namely, PP, UHMWPE, PS, and POM. These polymers are grouped together as they exhibit no significant changes during goniometry measurements, signifying their stability and resistance under experimental conditions related to wettability.

### 3.2. Surface Charge Determination

**Figure 2:**
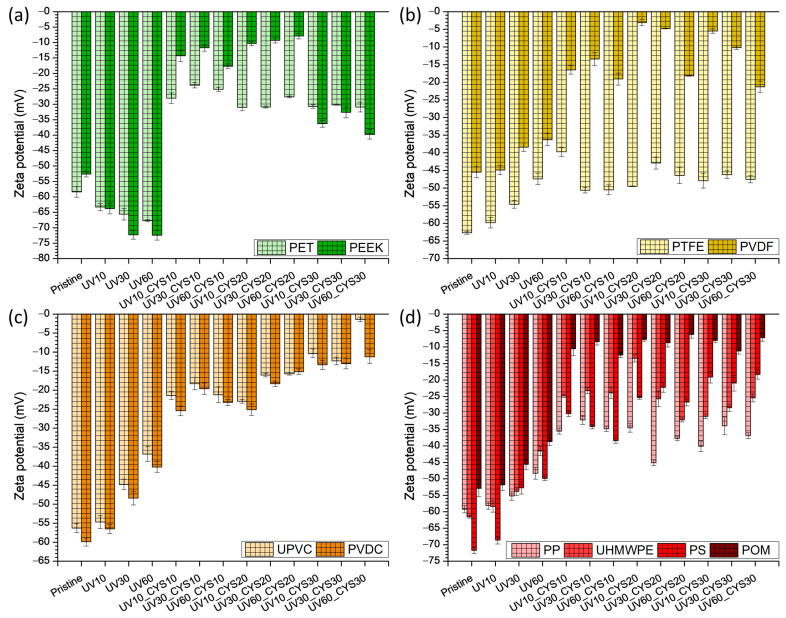
Comparison of zeta potential values of all pristine and functionalized polymers under study: (**a**) PET and PEEK; (**b**) PTFE and PVDF; (**c**) UPVC and PVDC; (**d**) PP, UHMWPE, PS, and POM.

The zeta potential values for all studied polymer foils before and after activation/modification are shown in Figure 2. Zeta potential is an indicator of the surface charge of materials in an electrolyte and is important in their interaction with other charged particles such as ions or biomolecules.

The zeta potential values for both PET and PEEK samples are presented in Figure 2a and demonstrate considerable changes following UV irradiation and subsequent grafting with cysteamine. UV radiation strongly affects the surface chemistry and charge of PET and PEEK polymers. Initially, the zeta potential values become more negative with increasing UV irradiation time, reflecting an increase in surface charge density. The increase in negative zeta potential can be attributed to the formation of oxygen-containing functional groups, such as hydroxyl (–OH) and carbonyl (–C=O) groups, on the polymer surface during UV irradiation. At the same time, the hydrophilicity of the surface increases after UV irradiation, which is reflected in a decrease in the contact angle values. The introduction of oxygen-containing functional groups increases the affinity of the polymer surface for water molecules, resulting in a more hydrophilic surface. In general, the dual effect of UV radiation—an increase in negative zeta potential and an increase in surface hydrophilicity—suggests an important activation and change in the chemical composition of the polymer surface. The increase in surface charge density and polarity facilitates interactions with aqueous solutions, while increased hydrophilicity can improve the biocompatibility and performance of polymers. Cysteamine grafting brings thiol (–SH) and amine (–NH_2_) functional groups to the polymer surface. The thiol groups in cysteamine can react with surface functional groups, such as carbonyl groups formed by UV irradiation, to form thioether (–S–C–) bonds [50]. Similarly, the amine groups in cysteamine can participate in reactions with surface functional groups to potentially form amide (–NH–C=O) or other nitrogen-containing bonds [15]. The presence of these sulfur- and nitrogen-containing functional groups contributes to a change in surface charge density and polarity as well as an increase in surface hydrophilicity [5,51]. Overall, the combined analysis of contact angle and zeta potential values suggests that the UV irradiation and cysteamine grafting treatments effectively change the surface properties of both PET and PEEK polymers. This is important and makes them more suitable for applications where enhanced interactions with aqueous solutions or biological systems are desired.

For the other polymer foils examined (Figure 2b–d), exposure to greater amounts of UV light caused a decrease in the zeta potential values, resulting in fewer negative values. This is in contrast to the PET and PEEK foils (Figure 2a). In Figure 2b,c, a small reduction in contact angle was detected for PTFE, PVDF, UPVC, and PVDC polymers. However, simultaneously, a shift towards fewer negative zeta potential values was observed. In a previous work [52], it was noted that oxygen groups developed on the surface of polyamide (PA 6), leading to a decrease in the negative values of the zeta potential. This article demonstrates that UV radiation impacts both the incorporation of new functional groups and the surface roughness, which significantly influences the change in zeta potential values. Upon being immersed in cysteamine solutions, the PVDF, UPVC, and PVDC polymers exhibited a pattern comparable to that of PET and PEEK. The zeta potential exhibited a quick transition towards less negative values, indicating changes in the chemistry and surface roughness, hence indicating the binding of cysteamine to the surface. Regarding the polymer PTFE, it is evident that changes in surface chemistry did not take place as a result of its resistance and lack of reactivity towards the 254 nm wavelength [53]. This is attributed to the presence of fluorine in its structure, unlike PVDF, which contains less fluorine.

While the contact angle values for PP, UHMWPE, PS, and POM films remained unaffected, their zeta potential values exhibited considerable variations during all stages of modification. According to this evidence, it seems that the primary factor that caused the change was the roughness of their surfaces [54]. Each of the four polymers shown in Figure 2d exhibited noticeable changes in zeta potential values upon exposure to cysteamine, with a pattern that closely resembled the preceding cases. To summarize, changes in surface chemistry, particularly those associated with the existence of charged groups, may result in changes in zeta potential without necessarily impacting the contact angle. However, changes in surface energy or roughness, which mostly impact the contact angle, may not always result in significant modifications in the zeta potential [55].

### 3.3. Elemental Surface Composition Analysis

To confirm our findings, we selected two representative samples, namely, UPVC and PEEK, for XPS analysis, the results of which are shown in Table 2. Table 2 presents the XPS element concentration data expressed as percentages of the total elements detected on the surface of both UPVC and PEEK samples. The elements include C (1s), O (1s), S (2s), and N (1s) for both polymers, as well as Cl (2p) for UPVC samples containing chlorine. These tests are important signs of changes in the surface chemical composition that occur after specific activation and chemical modification procedures. The impact of UV light on surface chemistry is obvious through the observed changes in elemental composition. The gradual rise in the amount of oxygen due to extended exposure to UV radiation highlights the significance of surface oxidation as a reaction to UV treatment [52,56]. The difference in the number of oxygen groups present on the surfaces of PEEK and UPVC samples exhibits a clear correlation with the results obtained from contact angle determination. Overall, PEEK has a greater tendency for the formation of oxygen groups compared to UPVC. Furthermore, the UPVC samples exhibit a higher percentage of chlorine content after prolonged exposure to UV radiation, suggesting the transfer of chlorine groups from the bulk to the surface. This redistribution is supported by prolonged UV exposure, which results in an enrichment of chlorine-containing molecules at the surface [57].

Moreover, the successful grafting of cysteamine onto the polymer surfaces is confirmed by the detection of nitrogen and sulfur elements, which are absent in pristine polymers and after UV radiation. The presence of nitrogen and sulfur following cysteamine grafting in all cases, compared to pristine or UV-irradiated samples, highlights the effectiveness of the grafting process in introducing desired functional groups onto the polymer surfaces. The impact of exposure time and cysteamine concentration on surface modification is notable. Higher exposure times and concentrations of cysteamine result in an increased presence of nitrogen and sulfur on the surface as percentages of the total elements detected. This correlation underscores the importance of optimizing exposure parameters and grafting conditions to achieve desired surface functionalization and enhance material properties. Moreover, the effectiveness of UV radiation in enhancing the success rate of grafting is shown by the remarkable results found after a 60 min UV exposure period. Extended exposure to UV light is believed to increase surface activation by creating more bonding sites, which, in turn, promotes more effective attachment of cysteamine molecules to the surfaces of the polymer [15,58].

**Figure 3:**
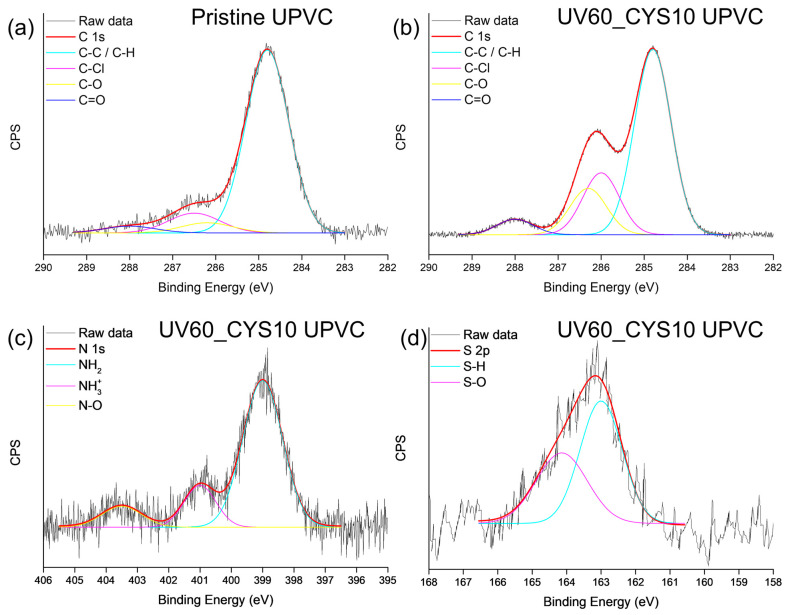
Fitted XPS spectra of pristine and modified UPVC samples, showing specific regions of interest. (**a**) C 1s spectrum of pristine sample, (**b**) C 1s spectrum of UV60_CYS10, (**c**) N 1s spectrum of UV60_CYS10, and (**d**) S 2p spectrum of UV60_CYS10.

Examples of the XPS spectral fittings for both pristine and modified UPVC are shown in Figure 3. The C 1s spectrum of pristine UPVC (Figure 3a) illustrates the chemical structure of the unmodified sample. A dominant peak at ~284.8 eV is labeled as C–C/C–H, reflecting the chemical environments typically present in the polymer backbone. A secondary peak at ~286.5 eV corresponds to C–Cl bonds, indicative of the chlorinated nature of UPVC. Minor peaks at ~286.2 eV and ~288.0 eV are attributed to C–O and C=O groups, respectively, likely resulting from surface oxidation processes during manufacturing or environmental exposure.

In the UV60_CYS10 sample, the C 1s spectrum (Figure 3b) reveals a reduction in the intensity of the C–C/C–H peak, attributed to the disruption of the polymer backbone during UV activation. UV irradiation generates surface radicals that facilitate subsequent chemical grafting, leading to a decrease in the detectable hydrocarbon content. The increased intensity of the C–Cl peak suggests a redistribution of chlorine within the polymer structure, potentially due to UV-induced chlorination reactions or surface restructuring. The slight decrease in the relative intensity of the C–O and C=O peaks reflects the addition of new surface elements, such as nitrogen and sulfur, which redistribute the overall percentage of elements detected by XPS. This does not imply the removal of oxidized species but rather a reduced relative contribution to the total spectrum due to the incorporation of cysteamine.

The N 1s spectrum (Figure 3c) reveals three distinct components corresponding to nitrogen-containing species. The peak at ~399 eV is attributable to –NH_2_ (amine) groups, representing the primary functional groups of grafted cysteamine. These amine groups are associated with surface interactions facilitated by UV-induced radical formation during surface activation. The component at ~401 eV corresponds to NH_3_^+^ (protonated amine) species, resulting from interactions between grafted cysteamine and residual oxidized groups, such as carboxyl or carbonyl functionalities on the polymer surface. Protonation of the amines introduces positively charged groups, influencing the observed zeta potential shift. A higher binding energy peak at ~403.5 eV is assigned to N–O bonds, indicating oxidative transformations of nitrogen during UV activation or subsequent exposure to oxygen. The presence of this component suggests that a portion of nitrogen underwent oxidation, forming nitrogen–oxygen bonds, likely as nitroso or nitro species. This highlights the reactivity of nitrogen under UV activation, contributing to the complexity of the modified surface chemistry.

**Figure 4:**
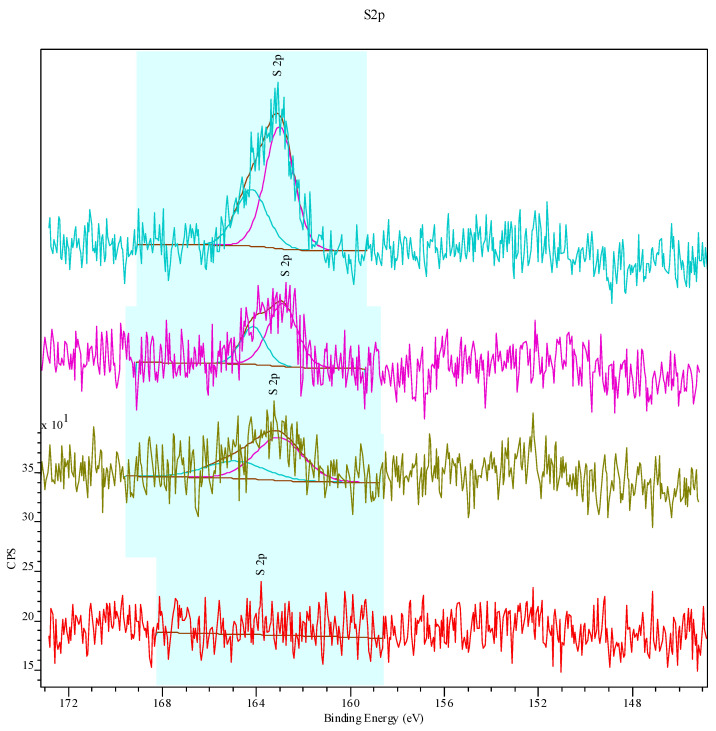
S (2p) sulfur spectrum on UPVC samples. From bottom: pristine (red), UV10_CYS10 (green), UV30_CYS10 (purple), and UV60_CYS10 (azure).

The S 2p spectrum of the UV60_CYS10 sample (Figure 3d) reveals two distinct peaks at ~163 eV and ~164 eV, corresponding to sulfur-containing species introduced through cysteamine grafting. The first component, observed at ~163 eV, is attributable to thiol (–SH) groups. These groups originate from cysteamine and indicate the presence of reduced sulfur species on the UPVC surface following the modification process. The second component, located at ~164 eV, is assigned to S–O bonds, representing oxidized sulfur species formed during the grafting process. UV activation and exposure to oxygen create a reactive environment that partially oxidizes thiol groups. The S–O species likely correspond to sulfenic acids (–SOH) or other intermediate oxidation states of sulfur. In Figure 4, the XPS spectra of modified UPVC with UV light for 10, 30, or 60 min followed by 10% cysteamine grafting shows the emergence of the S 2p peak.

### 3.4. Morphology of the Samples

**Figure 5:**
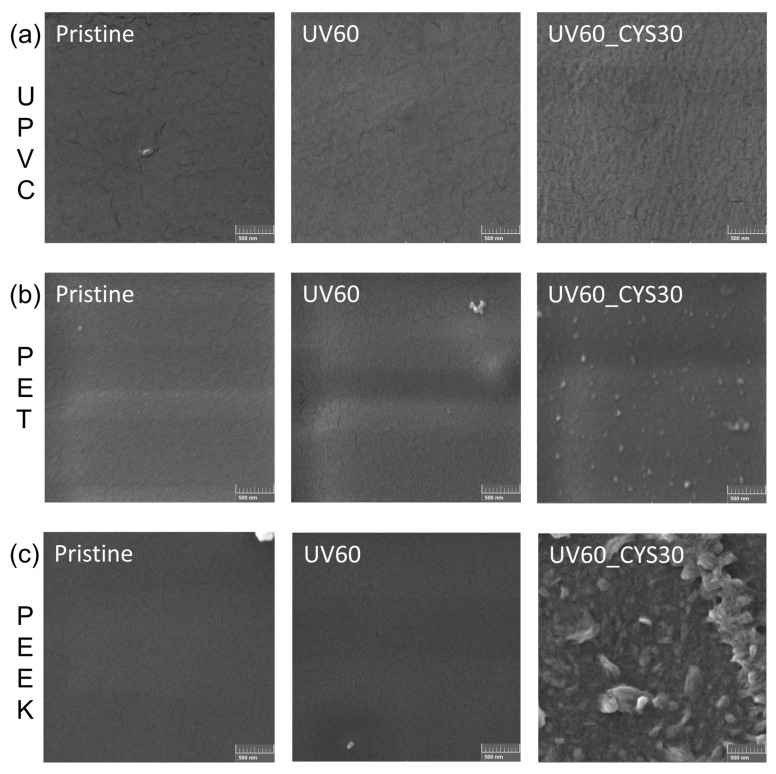
SEM micrographs of pristine, UV60, and UV60_CYS30 surfaces at 70k× magnification for (**a**) UPVC, (**b**) PET, and (**c**) PEEK. The accelerating voltage was 5 kV, and the scale bar represents 500 nm.

SEM micrographs (Figure 5) were obtained for representative samples, specifically UPVC, PET, and PEEK, to show the impact of UV activation and cysteamine grafting on surface morphology. These polymers were selected due to their diverse chemical compositions and surface properties.

As shown in the micrographs, the pristine UPVC surface (Figure 5a) appears relatively smooth with minor surface irregularities. Following UV activation for 60 min, noticeable roughening of the surface can be observed. This roughness likely results from photochemical reactions that introduce defects or modify the surface structure, enhancing its reactivity for subsequent grafting. In the case of the UV60_CYS30 sample, the surface shows further morphological changes, including increased texture and visible features that may correspond to the successful attachment of cysteamine functional groups. These modifications suggest that UV activation, combined with cysteamine grafting, visibly changes the surface morphology, which may improve the material’s interaction with microbial cells during antimicrobial testing. Surface roughness is a critical factor influencing antimicrobial efficacy. Studies have shown that nanostructured surfaces with specific topographies can mechanically disrupt bacterial cells, leading to increased bactericidal activity [59,60].

For PET samples (Figure 5b), the pristine PET surface appeared smooth and uniform, with minimal surface features, indicating no significant structural changes. After 60 min of UV activation (UV60), visible roughness and minor textural changes were observed on the PET surface. This roughening can be attributed to photochemical reactions induced by UV exposure, which introduce surface defects and increase reactivity for subsequent chemical grafting. Following cysteamine grafting (UV60_CYS30), the PET surface underwent further transformation, showing pronounced texturing and granular features. These changes suggest the successful attachment of cysteamine functional groups and a significant alteration of the surface morphology. Such modifications are expected to improve antimicrobial properties by enhancing bacterial interaction and reducing bacterial adhesion. When compared to UPVC UV60_CYS30, PET exhibits a more pronounced surface texture with finer and more evenly distributed features. In contrast, UPVC shows less defined morphological changes following UV activation and cysteamine grafting. This difference may reflect variations in the surface reactivity of the two materials, where PET appears more susceptible to UV-induced modification.

The surface morphology of PEEK foils before and after modification is shown in Figure 5c. The pristine PEEK surface appears smooth and featureless, similar to pristine PET and UPVC samples. This uniform surface confirms the absence of initial defects or structural irregularities. Following UV activation for 60 min (UV60), slight roughening of the PEEK surface is visible, but the changes are less pronounced compared to PET. The limited surface roughening could suggest lower susceptibility of PEEK to photochemical reactions under UV exposure. In contrast, the UV60_CYS30 sample demonstrates significant transformation, exhibiting a distinctly rough and textured surface with visible granular and irregular features. Compared to PET and UPVC, the changes in PEEK are more dramatic after cysteamine grafting, indicating strong chemical interactions facilitated by UV-induced activation. The pronounced roughening and granular structures on PEEK may result from higher grafting efficiency or deeper surface modifications. When compared to PET and UPVC, PEEK shows a unique progression in surface changes.

### 3.5. Algae Growth Resistance

**Figure 6:**
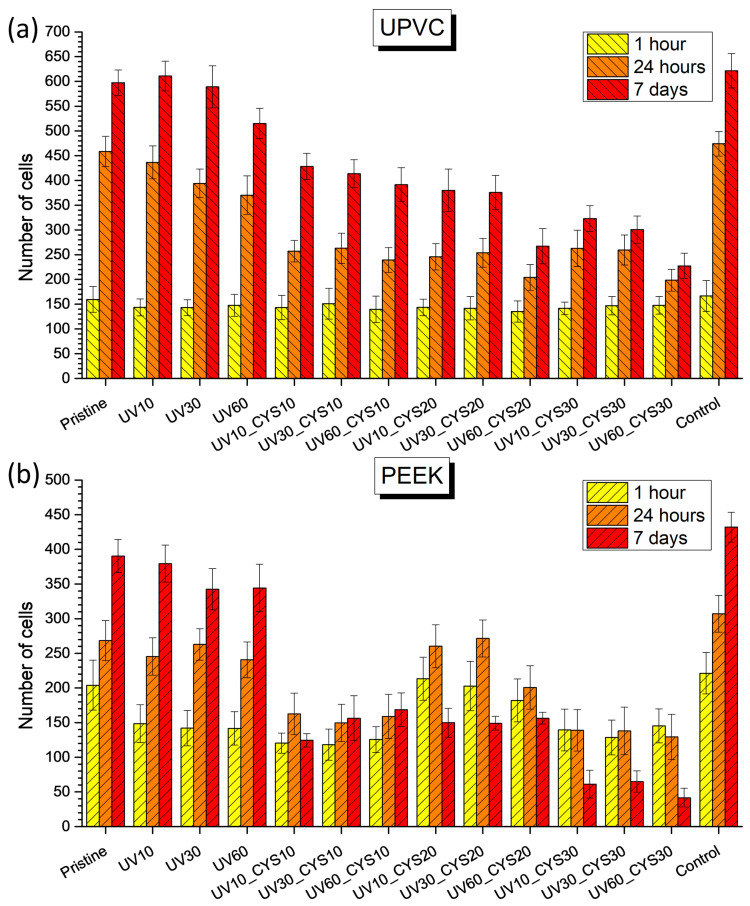
Results of *D. quadricauda* algal growth inhibition test for all samples of (**a**) UPVC and (**b**) PEEK.

The following experiments were performed to demonstrate the antimicrobial capabilities of the modified polymer surfaces. The primary objective of the experiment was to inhibit the proliferation of *D. quadricauda* algae, as illustrated in Figure 6. The pristine UPVC and PEEK samples served as a reference point to determine the effectiveness of surface modification procedures in preventing the growth of algae. The samples showed very little resistance to algal colonization, and the adhesion of algae increased dramatically as time went on. The lack of surface modification made the pristine surfaces vulnerable to fast algal growth, highlighting the need for efficient surface treatments for solving algae-related problems. While UV radiation alone had little to no effect on resistance to algae in most samples, there were notable exceptions among samples irradiated for 60 min (UV60). These samples exhibited slight improvements in algae resistance compared to the untreated pristine samples. The limited efficacy of UV radiation in enhancing resistance to algae suggests that additional surface modification techniques are necessary to achieve significant improvements in algal resistance. The findings indicated that samples that underwent shorter durations of UV exposure and were subsequently grafted with cysteamine (UV10_CYS10, UV30_CYS10, and UV60_CYS10) showed significant enhancements in their resistance to algae. The use of higher quantities of cysteamine led to more pronounced improvements, suggesting that the effect depends on the concentration. When the samples were exposed to radiation for longer periods of time and then treated with cysteamine (UV10_CYS20-UV60_CYS30), there was a noticeable improvement in their ability to resist algae. The effectiveness of cysteamine grafting was directly proportional to its concentration, as larger concentrations resulted in more significant enhancements in algae resistance. The control samples provided a reference point for evaluating the efficacy of surface modification techniques in preventing algal growth. As expected, the control samples exhibited higher levels of algal attachment and proliferation from the outset.

The effectiveness of cysteamine grafting in enhancing the resistance of UPVC and PEEK materials to algae is influenced by both the duration of UV exposure and the concentration of cysteamine. Prolonged UV exposure and higher concentrations of cysteamine generally result in more significant improvements in algal resistance. Thus, it is important to carefully adjust treatment conditions to achieve desired results effectively. This optimized approach leads to improved material performance and durability across various applications.

The anti-algal properties of cysteamine-modified polymer surfaces are primarily attributable to changes in surface wettability and the presence of reactive thiol (–SH) and amino (–NH_2_) functional groups. Surface wettability and surface charge influence the adhesion and colonization of algae, as hydrophobic and charge-altered surfaces have been shown to deter the initial settlement of algal cells, thereby reducing biofilm formation [61]. Thiol groups on cysteamine-modified surfaces can disrupt the algal biofilm matrix, particularly by disrupting extracellular polymeric substances (EPS) that are essential for biofilm integrity [62]. By weakening EPS, cysteamine reduces the structural stability of biofilms, which inhibits algal adhesion and growth. In addition, chemical modifications induced by cysteamine can change the ability of the surface to support algal colonization, further enhancing its antifouling properties [63].

### 3.6. Antibacterial Efficacy

**Figure 7:**
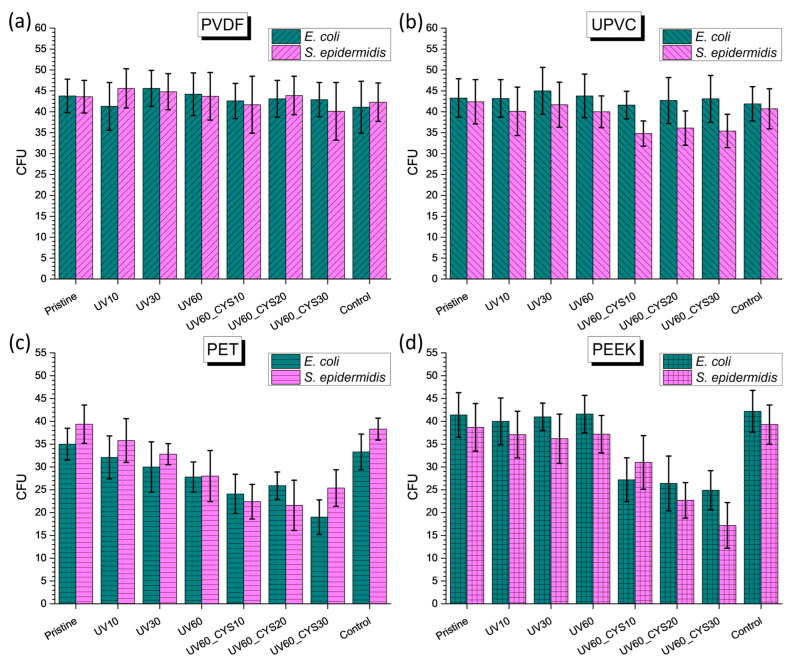
Numbers of CFUs of *E. coli* and *S. epidermidis* for samples of (**a**) PVDF, (**b**) UPVC, (**c**) PET, and (**d**) PEEK.

Based on previous results from the surface properties of the tested foils as well as their diversity, we decided to test some of them for antibacterial properties against *E. coli* and *S. epidermidis* (Figure 7). For the same purpose, we performed tests with a single concentration of cysteamine, specifically 30 wt. %. The results for the modified PVDF samples are shown in Figure 7a. At first observation, it is evident that there is no reduction in the number of colonies for either bacterial strain in these samples. Although changes in zeta potential values were detected, PVDF did not exhibit any antibacterial effectiveness. Therefore, we attribute the changes in zeta potential to a modification in surface morphology rather than surface chemistry. The differences in activity between the tested polymers may also be influenced by the structural differences between Gram-negative (*E. coli*) and Gram-positive (*S. epidermidis*) bacteria, as Gram-negative bacteria are often more resistant due to their outer membrane. Conversely, Figure 7b demonstrates that UPVC samples exhibited at least a minimal impact on S. epidermidis after cysteamine attachment. Nevertheless, the current amount of cysteamine is not enough to induce a substantial reduction in bacteria.

Considerably interesting findings can be observed for PET (Figure 7c) and PEEK (Figure 7d) samples. Following exposure to UV irradiation alone, there is no notable change in bacterial reduction except for a modest effect on the PET film, which is not statistically significant. However, upon the subsequent attachment of cysteamine, there is a decrease in the number of bacteria. When cysteamine is attached to PET foils, changes in colony counts are noticeable for all UV irradiation times applied. This impact is evident in both bacterial strains. Sample UV60_CYS30 of PET exhibits a significant decrease of 45.7% in *E. coli* and a 35.5% decrease in *S. epidermidis*. When examining PEEK samples, a noticeable pattern occurs as the exposure period increases and cysteamine is subsequently grafted. According to the XPS data, an increased concentration of cysteamine leads to a higher reduction in bacterial colonies. The PEEK sample UV60_CYS30 demonstrates the greatest results, with a 39.9% reduction in *E. coli* colonies and a 55.6% reduction in *S. epidermidis* colonies. In summary, the results suggest that the combination of UV irradiation and cysteamine grafting enhances the antibacterial properties of PET and PEEK samples. The effectiveness of this approach is influenced by factors such as UV irradiation time, cysteamine concentration, and surface chemistry.

The antibacterial activity of cysteamine-modified polymer surfaces is attributed to the presence of thiol (–SH) and amino (–NH_2_) functional groups, which are important in inhibiting bacterial growth and destabilizing biofilms. The thiol group interacts with disulfide bonds in bacterial proteins and the extracellular matrix of biofilms. Disulfide bonds are essential for maintaining the structural integrity of bacterial cell walls and biofilms. By disrupting these bonds, cysteamine weakens the bacterial cell structure, leading to destabilization and inhibition of bacterial growth [49]. Additionally, the amino groups on cysteamine-modified surfaces facilitate electrostatic interactions with the negatively charged bacterial membrane. These interactions increase membrane permeability, resulting in leakage of intracellular components and ultimately bacterial cell death [64].

In comparison, our earlier study [22] highlights distinct trends in antibacterial performance for chemically modified PEEK and PET samples. Short UV irradiation times (e.g., 10 min) followed by grafting with alanine or leucine were sufficient to achieve reductions in *E. coli* colonies. This trend highlights the sensitivity of Gram-negative bacteria, such as *E. coli*, to hydrophilic surfaces and amino group interactions. In contrast, longer UV irradiation times (e.g., 60 to 120 min) were required to maximize the antibacterial effect of curcumin, especially against *S. epidermidis*. This dependency likely arises from curcumin’s reliance on surface oxidation and roughness to enhance biofilm disruption, which is particularly effective against Gram-positive bacteria.

The current study demonstrates that cysteamine exhibits a more uniform response across both Gram-positive and Gram-negative bacteria compared to alanine, leucine, and curcumin. UV irradiation time was found to be an important factor in enhancing antibacterial performance, with longer exposure resulting in better bacterial reductions. The consistent antibacterial activity of cysteamine-modified PET and PEEK, regardless of bacterial type, shows its versatility compared to the bacteria-specific trends observed in the earlier study. These findings indicate that cysteamine’s dual functional groups (thiol and amino) enable both biofilm destabilization and electrostatic interactions, making it a promising grafting agent for antibacterial surface modification across a variety of polymer substrates.

## 4. Conclusions

The findings from this study offer useful knowledge about the modification of polymer foils’ surfaces by UV activation and cysteamine grafting. The study also explores the resulting changes in surface properties and the effectiveness of the modified surfaces in preventing bacterial growth. Noticeable changes in surface characteristics were noted after both exposure to UV light and the application of chemical grafting to the polymer foils. Surface wettability, as evidenced by contact angle measurements, underwent notable changes upon UV activation and cysteamine grafting. The introduction of cysteamine led to changes in surface hydrophilicity, with increased concentrations resulting in heightened surface wettability. These changes were attributable to the formation of reactive sites on the polymer surface during UV irradiation, facilitating the subsequent attachment of cysteamine molecules. Zeta potential measurements provided further insights into the surface charge of the polymer foils. UV irradiation resulted in changes in surface charge density, while cysteamine grafting further modified surface chemistry, as indicated by shifts in zeta potential values. The presence of the elements nitrogen and sulfur following cysteamine grafting confirmed the successful introduction of desired functional groups onto the polymer surfaces, highlighting the effectiveness of the grafting process.

XPS analysis revealed changes in surface chemical composition following UV activation and cysteamine grafting. Increased oxygen content, particularly in PEEK samples, was observed after UV exposure, indicating surface oxidation. Additionally, the detection of chlorine enrichment on UPVC surfaces suggested the migration of chlorine groups from the bulk to the surface upon UV irradiation. Successful grafting of cysteamine was confirmed by the presence of nitrogen and sulfur elements, absent in pristine and UV-irradiated samples.

SEM analysis confirmed that UV activation introduced surface roughness, while cysteamine grafting further transformed the morphology, particularly in PET and PEEK, where pronounced texturing and granular features were observed. These surface changes likely contribute to improved antimicrobial efficacy by enhancing bacterial interactions and reducing adhesion.

Antimicrobial testing demonstrated the enhanced resistance of polymer foils to algal growth and bacterial colonization following surface modification. While UV irradiation alone had a limited impact on algae inhibition, the subsequent attachment of cysteamine significantly improved resistance, with higher concentrations yielding greater enhancements. PET and PEEK samples showed notable reductions in bacterial colonies following cysteamine grafting, with effectiveness influenced by UV irradiation time.

In conclusion, the combination of UV activation and cysteamine grafting presents a promising approach for enhancing the surface properties and antibacterial effectiveness of polymer foils. Optimizing treatment parameters such as UV irradiation time and cysteamine concentration allows tailored modifications that can achieve desired surface functionalities. These findings contribute to the development of polymer materials with improved performance and durability across various applications, from biomedical devices to packaging materials.

## Figures and Tables

**Table 1:** List of samples and their marking (CYS = cysteamine).

Sample	Description
Pristine	Pristine (unmodified foil)
UV10	Polymer foil activated by UV radiation for 10 min
UV30	Polymer foil activated by UV radiation for 30 min
UV60	Polymer foil activated by UV radiation for 60 min
UV10_CYS10	Polymer foil activated by UV radiation for 10 min and grafted with 10 wt. % CYS
UV30_CYS10	Polymer foil activated by UV radiation for 30 min and grafted with 10 wt. % CYS
UV60_CYS10	Polymer foil activated by UV radiation for 60 min and grafted with 10 wt. % CYS
UV10_CYS20	Polymer foil activated by UV radiation for 10 min and grafted with 20 wt. % CYS
UV30_CYS20	Polymer foil activated by UV radiation for 30 min and grafted with 20 wt. % CYS
UV60_CYS20	Polymer foil activated by UV radiation for 60 min and grafted with 20 wt. % CYS
UV10_CYS30	Polymer foil activated by UV radiation for 10 min and grafted with 30 wt. % CYS
UV30_CYS30	Polymer foil activated by UV radiation for 30 min and grafted with 30 wt. % CYS
UV60_CYS30	Polymer foil activated by UV radiation for 60 min and grafted with 30 wt. % CYS

**Table 2:** Atomic concentration of elements (in %) obtained by XPS analysis for all PEEK and UPVC samples.

UPVC	C (1s)	Cl (2p)	O (1s)	N (1s)	S (2p)	PEEK	C (1s)	O (1s)	N (1s)	S (2p)
Pristine	83.3	9.0	7.7	-	-	Pristine	84.6	15.4	-	-
UV10	77.4	14.8	7.8	-	-	UV10	81.2	18.8	-	-
UV30	74.8	17.0	8.2	-	-	UV30	77.3	22.7	-	-
UV60	71.2	18.8	10.0	-	-	UV60	76.9	23.1	-	-
UV10_CYS10	77.7	13.2	7.5	1.0	0.6	UV10_CYS10	80.8	13.1	3.2	2.9
UV30_CYS10	77.2	13.0	7.2	1.7	0.9	UV30_CYS10	79.7	13.0	3.6	3.7
UV60_CYS10	76.8	13.1	7.0	2.0	1.1	UV60_CYS10	79.3	12.1	4.0	4.6
UV10_CYS20	75.4	15.6	7.2	1.1	0.7	UV10_CYS20	76.9	15.4	3.8	3.9
UV30_CYS20	75.1	15.1	7.1	1.7	1.0	UV30_CYS20	78.1	12.5	4.5	4.9
UV60_CYS20	75.0	15.2	6.8	1.9	1.1	UV60_CYS20	78.0	12.8	4.3	4.9
UV10_CYS30	71.3	17.6	7.6	1.9	1.6	UV10_CYS30	77.1	15.1	3.7	4.1
UV30_CYS30	70.7	17.3	7.6	2.6	1.8	UV30_CYS30	76.0	12.5	5.1	6.4
UV60_CYS30	70.8	17.1	7.4	2.7	1.0	UV60_CYS30	76.0	12.7	5.2	6.1

## Data Availability

The data supporting the findings of this study are available from Zenodo under the following DOI: 10.5281/zenodo.14442314.
